# Revealing Novel Source of Breast Cancer Inhibitors from Seagrass *Enhalus acoroides*: In Silico and In Vitro Studies

**DOI:** 10.3390/molecules29051082

**Published:** 2024-02-29

**Authors:** Yan Wisnu Prajoko, Faqrizal Ria Qhabibi, Timothy Sahala Gerardo, Kanandya Kizzandy, Krisanto Tanjaya, Sebastian Emmanuel Willyanto, Happy Kurnia Permatasari, Reggie Surya, Nelly Mayulu, Nurpudji Astuti Taslim, Raymond Rubianto Tjandrawinata, Rony Abdi Syahputra, Trina Ekawati Tallei, Apollinaire Tsopmo, Bonglee Kim, Rudy Kurniawan, Fahrul Nurkolis

**Affiliations:** 1 Department of Surgical Oncology, Faculty of Medicine, Diponegoro University, Semarang 50275, Indonesia; 2 Faculty of Medicine, Brawijaya University, Malang 65145, Indonesia; 3 Department of Food Technology, Faculty of Engineering, Bina Nusantara University, Jakarta 11480, Indonesia; 4 Department of Nutrition, Faculty of Health Science, Muhammadiyah Manado University, Manado 95249, Indonesia; 5 Faculty of Medicine, Hasanuddin University, Makassar 90245, Indonesia; 6 Department of Biotechnology, Faculty of Biotechnology, Atma Jaya Catholic University of Indonesia, Jakarta 12930, Indonesia; 7 Department of Pharmacology, Faculty of Pharmacy, Universitas Sumatera Utara, Medan 20155, Indonesia; 8 Department of Biology, Faculty of Mathematics and Natural Sciences, Universitas Sam Ratulangi, Manado 95115, Indonesia; 9 Food Science and Nutrition Program, Department of Chemistry, Carleton University, 1125 Colonel by Drive, Ottawa, ON K1S 5B6, Canada; 10 Department of Pathology, College of Korean Medicine, Kyung Hee University, Seoul 02447, Republic of Korea; 11 Korean Medicine-Based Drug Repositioning Cancer Research Center, College of Korean Medicine, Kyung Hee University, Seoul 02447, Republic of Korea; 12 Department of Biological Sciences, Faculty of Sciences and Technology, State Islamic University of Sunan Kalijaga (UIN Sunan Kalijaga), Yogyakarta 55281, Indonesia; fahrul.nurkolis.mail@gmail.com

**Keywords:** seagrass, anticancer, natural products, antioxidants, breast cancer, metabolites

## Abstract

*Enhalus arcoides* is a highly beneficial type of seagrass. Prior studies have presented proof of the bioactivity of *E. acoroides*, suggesting its potential to combat cancer. Therefore, this study aims to delve deeper into *E. acoroides* bioactive molecule profiles and their direct biological anticancer activities potentials through the combination of in-silico and in-vitro studies. This study conducted metabolite profile analysis on *E. acoroides* utilizing HPLC-ESI-HRMS/MS analysis. Two extraction techniques, ethanol and hexane, were employed for the extraction process. Furthermore, the in-silico study was conducted using molecular docking simulations on the HER2, EGFR tyrosine kinase and HIF-1α protein receptor. Afterward, the antioxidant activity of *E. acoroides* metabolites was examined to ABTS, and the antiproliferative activity was tested using an MTT assay. An in-silico study revealed its ability to combat breast cancer by inhibiting the HER2/EGFR/HIF-1α pathway through molecular docking. In addition, the MTT assay demonstrated that higher dosages of metabolites from *E. acoroides* increased the effectiveness of toxicity against cancer cell lines. Additionally, the study demonstrated that the metabolites possess the ability to function as potent antioxidants, effectively inhibiting a series of carcinogenic mechanisms. Ultimately, this study showed a new approach to unveiling the *E. acoroides* metabolites’ anticancer activity through inhibiting HER2/EGFR/HIF-1α receptors, with great cytotoxicity and a potent antioxidant property to prevent a carcinogenic cascade.

## 1. Introduction

Seagrasses are compressive plants in coastal areas. The plants provide many functions for the marine biota, from providing breeding habitats for a variety of marine species to serving as maintenance plants for numerous marine species, including rare species. Seagrasses typically inhabit sandy substrates, silty sand, mud, and coral debris in shallow marine settings, saltwater estuaries, persistent pools of water, and open water during low tide. Seagrass is frequently utilized for composting, netting, and as animal feed in diverse areas. In addition, seagrasses are utilized as a cosmetic ingredient, as well as in medicine and other medicinal applications, particularly in industrialized nations [1]. Seagrasses have historically been employed in traditional medicine to treat a range of medical conditions, such as fevers, gastrointestinal problems, muscular discomfort, injuries, and dermatological disorders. Additionally, they are utilized as a treatment for radiation-related burns and as a sedative for infants. Aside from their medicinal importance, these seagrasses are utilized in several industries such as basket weaving, salt manufacturing, mattress stuffing, thatched roof construction, fertilizer manufacture, paper materials for delicate item transportation, and nitrocellulose manufacturing, among other uses. *Enhalus arcoides* is a highly beneficial type of seagrass [2].

Seagrasses, an integral component of coastal ecosystems, play a pivotal role in maintaining marine biodiversity. There are about 72 species of 12 genera of seagrasses throughout the world in a wide variety of habitats. Among these, *E*. *acoroides* (L.f.) Royle belongs to Enhalus, a monotypic marine genus in the family Hydrocharitaceae, particularly prevalent in the coastal regions of Southeast Asia and is easily found in Indonesia [3,4]. *E. Acoroides* is notable for its distinct physical characteristics and ecological importance as it provides a vital environment for a wide range of marine creatures [5]. However, beyond its ecological role, emerging studies suggest that *E*. *acoroides* harbors bioactive compounds with potential medicinal properties, opening new avenues for exploration in the realm of natural product-based drug discovery [6]. Seagrasses, particularly *E. arcoides,* are renowned for their ability to produce a diverse range of secondary metabolites that serve as defense mechanisms in challenging conditions. These active compounds, including polyphenols, terpenoids, and halogenated compounds, are synthesized from the seagrass and possess numerous beneficial properties such as antifungal, anti-inflammatory, antibacterial, antiviral, antidiabetic, antimalarial, and antioxidant effects. Moreover, they exhibit aging and cytotoxic properties. Notably, these species are effective in preventing a wide array of diseases in humans [1,6,7]. Nevertheless, there is currently limited scientific investigation regarding the efficacy of *E. acoroides* in combating cancer, particularly in relation to breast cancer. This area of research needs extensive discovery of innovative compounds derived from natural sources for the purpose of treatment.

Prior studies have presented proof of the bioactivity of *E. acoroides*, suggesting its potential as a viable candidate for pharmacological research and diverse other applications. The discovery of novel chemicals exhibiting potent capabilities, especially in terms of their ability to combat cancer, has great potential, as emphasized in previous research [8]. Against this background, our research aims to delve deeper into *E*. *acoroides* bioactive molecule profiles and their direct biological anticancer activities potentials. Through an integrated approach that combines advanced in silico and in vitro methodologies, we seek to identify and characterize specific bioactive compounds within *E*. *acoroides* with anti-breast cancer properties. Furthermore, in this research, maceration was used with two different solvents, each possessing different polarities—specifically, n-hexane (nonpolar) and ethanol (polar). This approach aimed to reveal various compounds based on their varying degrees of polarity. By profiling the metabolites, anticancer, and antioxidant properties of *E*. *acoroides*, this research not only aligns with the broader exploration of natural sources for cancer therapeutics but also holds promise for uncovering novel leads in the fight against breast cancer.

## 2. Results

### 2.1. List of Compounds after Metabolomic Profiling

The metabolite profiles of Seagrass *Enhalus acoroides* are successfully obtained and analyzed using non-targeted metabolomic profiling HPLC-ESI-HRMS/MS analysis (Table 1), using two different extraction solvents: ethanol and hexane. The samples from the extraction that are found are shown in Table 1. Two samples were obtained from Seagrass *E*. *acoroides*, EAE (*E. acoroides*—ethanol), and EAH (*E. acoroides*—hexane), five different compounds were obtained from EAE, and five different compounds were obtained from EAH. The type of compounds of the metabolites obtained in the study depends on the solvent that is used in the maceration.

### 2.2. Pa Score, Toxicity Prediction, Drug Likeness and Network Pharmacology Analysis

To clarify the targeting pathway at the molecular docking stage, Pa score, toxicity prediction, drug likeness and network pharmacology analysis were carried out on *E. acoroides* extract compounds with target proteins and breast cancer gene proteins, as presented in Table 2. Based on the data analysis presented in Table 2, there are four compounds that have the potential to become drug candidates targeting anti-breast cancer, including compounds C2, C3, C8, and C9. These four compounds have potential value seen from the Pa value against breast cancer-related receptors HIF1A expression and Chlordecone reductase and followed by a predicted LD_50_ value of >1000 or toxicity class of >4, and fulfill the Lipinski Rule with the information “Accepted” as shown in Table 2.

**Figure 1:**
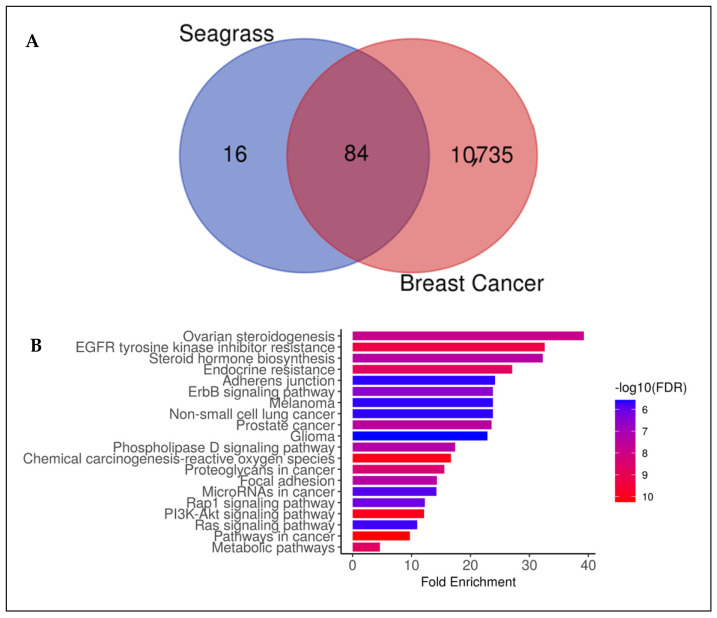

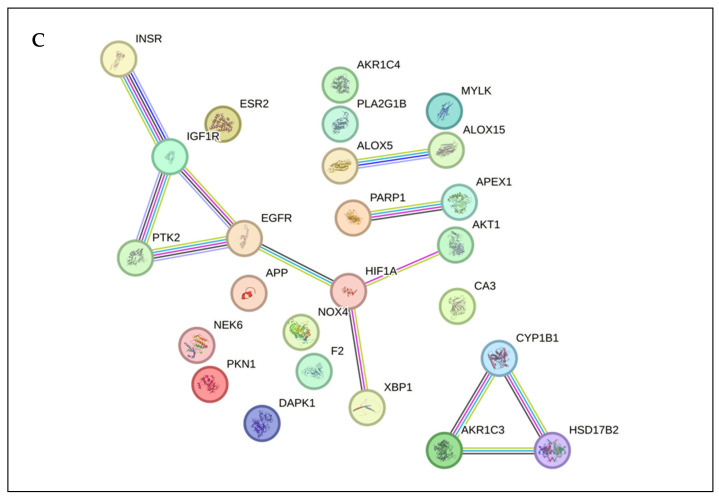
Network Pharmacology *E. acoroides* Extract against breast cancer. (**A**) Venn diagram showing shared targets between *E. acoroides* Extract and genes associated with breast cancer. (**B**) Annotation of gene ontology biological processes for *E. acoroides* Extract targets (false discovery rate or FDR < 0.90). (**C**) Protein–protein interaction (PPI) of *E. acoroides* Extract targets in breast cancer.

To find central receptors that play a role in cancer signaling, especially breast cancer, network pharmacology analysis was carried out. In the analysis of disease-related targets and targets from Seagrass *E. acoroides* Extract mapped on the Venn diagram Figure 1A, it was found that the corresponding target intersections from seagrass and breast cancer were 84 genes and proteins. Advanced analysis of interactions between target proteins obtained from Seagrass *E. acoroides* Extract and their relationship to breast cancer produced several possible signals in cancer management, such as EGFR tyrosine kinase, pathways in cancer, metabolic pathways, and chemical carcinogenesis-ROS (Figure 1B). In network pharmacology (Figure 1B), it shows the central receptor related to cancer, namely EGFR as presented in Table 3.

In Table 3, EGFR is observed as a prospective target receptor of *E. acoroides*, and its ability to interact with the HIF-1Alpha receptor is shown. It was observed that PIK3CA was also associated with EGFR, this implies that *E. acoroides* Extract is also involved in pathways such as PI3K/AKT and HIF-1Alpha signaling which were recognized as cancer makers. Several signaling pathways were also observed that allow for further studies, such as the HIF-1 signaling pathway, EGFR tyrosine kinase inhibitor resistance (Cancer), ERK and HER signaling (cancer), and PI3K signaling (cancer) which have the potential to be continued with molecular docking as a receptor. Therefore, three potential receptors were selected to continue the molecular docking simulation, HIF-1A, EGFR tyrosine kinase, and HER2. For the data, the values of degree, betweenness centrality, and closeness centrality can be seen in Supplementary Materials.

### 2.3. Docking Potency of Compounds Found in EAE and EAH 

The molecular docking simulation of the drug targets that are used is shown in Table 4. The potency of identified compounds of Seagrass *E. acoroides* that are used as compounds for molecular docking with HER2, EGFR tyrosine kinase and HIF-1α receptors as the drug target, as shown in Table 4. Doxorubicin and Talazoparib, a chemotherapy agent and a cancer drug, were used as the control compounds with affinity values shown in Table 4. All compounds (C2, C3, C8, and C9) clearly have good binding affinity values (better than Doxorubicin and Talazoparib control affinity values as threshold) on the three receptors. 

The performance of the substance found in EAE and EAH on HER2, EGFR tyrosine kinase and HIF-1α protein can be determined by the binding activity of the named substance to block the signal binding to receptors expressed in Table 5. The performance of such substances can be explained by the strength and amount of amino acid binding that occurs which prevents signal binding towards receptors. While the amount of amino acid binding may explain the utilization flexibility of such a substance, the strength of the binding through various chemical bonds, namely hydrogen bonds might explain the substance affinity. Most of the substances, found in each EAE (C2, C3) and EAH (C8, C9) expressed hydrogen binding against amino acids which plays a role in HER2/EGFR tyrosine kinase/HIF-1α signaling pathways. Thus, this explains the varying degrees of docking activity of each substance in relation to its chemical form and activity.

### 2.4. Scavenging Activity, Anticancer Capacity, and Safety of EAE and EAH

**Figure 2:**
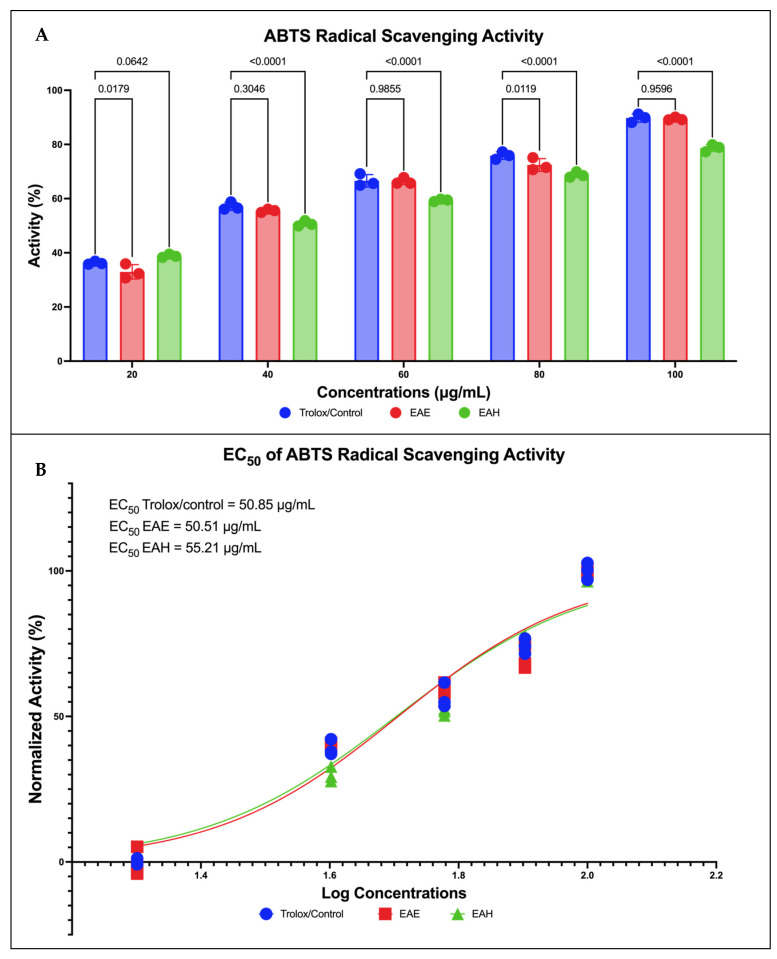
Antioxidant capabilities of Seagrass *E. acoroides*. (**A**) Two-way ANOVA analysis of various concentrations of Seagrass *E. acoroides* extract. (**B**) EC_50_ of ABTS inhibition activity.

Through two-way ANOVA analysis comparing the radical scavenging activity of EAE and EAH compared to Trolox as control, it can be conferred that EAE has no significant differences compared to Trolox in 20 µg/dL and 40 µg/dL concentrations where it can be seen in Figure 2A, that it has slightly lower radical scavenging activity compared to the control group. Although in the other concentrations, there is no significant difference between EAE and Trolox which can be inferred that EAE’s scavenging activity is mostly non-inferior compared to Trolox as the control group. On the other hand, the performance of EAH in all concentrations compared to Trolox is found to be statistically significant except in the concentration of 20 µg/dL. The activity percentage of EAH compared to Trolox was found to be inferior in all concentrations except in 20 µg/dL, which from this finding, it can be inferred that the ROS scavenging performance of EAH is inferior compared to Trolox as a control group.

Similar findings were also noted in the EC_50_ found in each Trolox, EAE, and EAH group expressing each substance’s efficacy. EAE has the lowest EC_50_ among the three groups, with 50.51 µg/dL, followed by Trolox at 50.85 µg/dL, and EAE at 55.21 µg/dL. The EC_50_ analysis in Figure 2B inferred the same findings as found in Figure 2A where EAE has superior potency in terms of radical scavenging activity compared to the control group and EAH group. Furthermore, further global ANOVA was carried out and it was found that there were significant differences between treatment groups in the ABTS inhibition test (*p* < 0.0001).

The LD_50_ values of samples obtained by Seagrass *E. acoroides* extract on breast cancer cell lines and normal epithelial cell lines, with doxorubicin as a control sample, are shown in Table 6. From these data, it can be concluded that EAE and EAH samples have LD_50_ values on breast cancer cells far from being potential or strong in killing MCF-7, and MDA-MB-231 breast cancer cells, but also samples (EAE and EAH) require a much higher dose than doxorubicin in order to kill breast cancer cells. More interestingly, when further analysis was carried out, the results found single commercial compounds Luteolin (C2) and Thalassiolin C (C8) which were also observed in Seagrass *E. acoroides*. The results showed that they were more potent than in the form of whole extract (LD_50_ C2 and C8 < EAH and EAE). As an additional note, C3 and C9 cannot be tested at this stage due to research limitations and it is hoped that this can be undertaken in the future to complement these results. In addition, the LD_50_ of normal MCF-10A cells shows a higher value than the control drug and is >1500 μg/mL, so it is considered safe for further use or consumption as an alternative chemotherapy agent for breast cancer. This in vitro study validates the in silico molecular docking study which shows that Seagrass *E. acoroides* has great potential as an anti-breast cancer agent.

**Figure 3:**
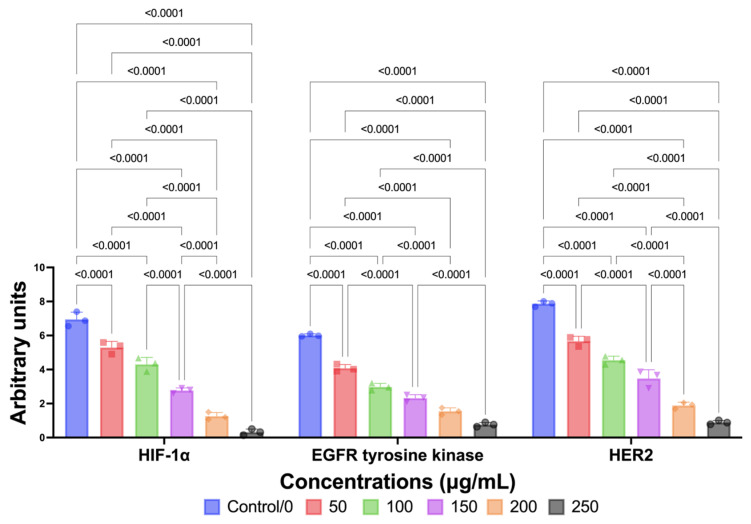
Downregulation of HIF-1A, EGFR tyrosine kinase, and HER2 by Seagrass EAE.

In line with in silico, in vitro studies showed that a significant pattern of reduction or suppression of HIF-1A, EGFR tyrosine kinase, and HER2 was observed in MCF-7 cancer cells given EAE treatment (*p* < 0.05; Figure 3). EAE significantly suppressed HIF-1A, EGFR tyrosine kinase, and HER2 compared to controls not given EAE. The choice of EAE alone (without EAH) in this assessment was due to the assessment of antioxidant and antiproliferative activity with the maximum potential being EAE compared to EAH (Figure 3). Furthermore, further global ANOVA was carried out and it was found that there were significant differences between treatment groups for each maker (*p* < 0.0001). Surprisingly, this certainly confirms the in silico results which also show that Seagrass *E. acoroides* has superior potential in fighting HIF-1A, EGFR tyrosine kinase, and HER2 as manifestation markers of breast cancer.

## 3. Discussion

Nature comprises diverse species that actively generate both primary and secondary metabolites. Secondary metabolites are organic compounds synthesized through a metabolic pathway and are not directly associated with plant growth and development [9,10]. Marine organisms are among the contributors to secondary metabolic sources and their phytochemical derivatives. The comprehensive investigation of phytochemical composition in marine organisms was conducted extensively, with the exception of certain minor taxonomic classes found in seagrass, which have not been thoroughly examined. Seagrass thrives most abundantly in tropical oceans, making them the most productive region for this type of vegetation. Southeast Asia is renowned for its rich seagrass diversity, hosting 24 out of the 60 identified seagrass species. These species thrive in the warm waters of Indonesia, the Philippines, Thailand, and Vietnam [10,11]. *E. acoroides* is a seagrass species primarily found in Indonesia, among the 24 species of seagrass. This study conducted metabolite profile analysis on *E. acoroides* utilizing HPLC-ESI-HRMS/MS analysis. Two extraction techniques, ethanol and hexane, were employed for the extraction process. In this work, the extraction of both EAE and EAH yielded a total of five distinct types of metabolite components. De Vincenti et al. conducted phytochemical profiling on the same species using liquid chromatography/mass spectrometry (LC/MS). The results revealed that *E. acoroides* is rich in flavonoids such as apigenin, luteolin, three derivatives of kaempferol, and azelaic acid. In addition, independent studies have demonstrated the presence of phenolic metabolites, such as caffeic, apigenin, and luteolin with sulfate, in different seagrass species, including *E. acoroides* [12,13].

**Figure 4:**
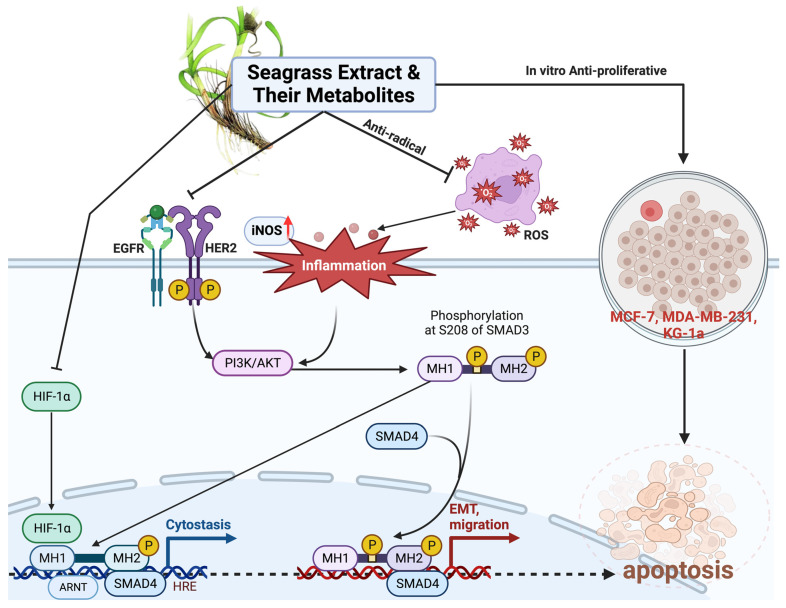
Mechanism of action from Seagrass *E. acoroides* in Combating Breast Cancer. Created with BioRender.com Premium License by Fahrul Nurkolis (https://app.biorender.com, accessed on 5 January 2024).

The study involved docking analyses on the metabolite components of both EAE and EAH in order to assess the potential of these current metabolite components. The receptors targeted for binding in this investigation are HIF-1α, EGFR tyrosine kinase, and HER2, with doxorubicin and talazoparib selected as the control substances (Figure 4). Doxorubicin, also referred to as Adriamycin, is a chemotherapy medication widely utilized in the treatment of different forms of cancer. It effectively triggers cell apoptosis through a range of intracellular mechanisms, including inhibiting Topoisomerase II, evicting histones, generating reactive oxygen species, and overproducing ceramide (Figure 4) [14,15]. The molecular docking analysis in this study revealed that nearly all metabolite constituents obtained from *E. acoroides* utilizing ethanol and hexane exhibited a greater affinity for the target receptors in comparison to the control, doxorubicin and talazoparib. Meanwhile, the metabolite components obtained using ethanol extraction (EAE) demonstrated that two out of the five components had a greater affinity for binding compared to the control. The negative sign on the affinity value or ΔG (kcal/mol) indicates an exergonic reaction in the binding process between the metabolite component and the target receptor [16]. There are four metabolite components (C2, C3, C8, C9) that have striking affinity values for the HIF-1α, EGFR tyrosine kinase, and HER2 receptors when compared with other metabolite components from the results of the two extraction methods and control (drugs). These four metabolites include luteolin, Thalassiolin C, *O*-caffeoyl-*O*-coumaroyl tartaric acid, Luteolin, and luteolin-*O*-sulphate.

Luteolin belongs to a group of flavonoids that are found abundantly in *E. acoroides*. The study conducted by Mabrouk et al. showed that luteolin has efficacy as an anticancer compound. It was tested in-vitro using the non-carcinogenic endothelial cell line hCMEC. Cell viability was considerably decreased by luteolin at doses of 2.5 μg/mL and 12.5 μg/mL, by 35% and 72%, respectively [17]. A different in vitro study reported that luteolin treatment of MG63 and MG64 cells inhibited cell growth by upregulating the expression of the Bax protein, which in turn caused the expression of BCL-2 and caspase-3 to be downregulated. In addition, tests on the T-cell Lymphoma Cell Line CCRF-CEM showed induction of arrest in the S phase and apoptosis through increased expression of Bax, caspase-9, and cascade [2]. Research on the anticancer properties of the metabolite components of *E. acoroides* was also carried out by Ahmed et al. who showed that metabolite compounds from *E. acoroides* have promising anticancer potential, as they were tested on various types of cell lines (Figure 4). The MTT (3-[4,5-dimethylthiazol-2-yl]-2,5 diphenyl tetrazolium bromide) assay demonstrated that elevating the concentration of the extract led to an augmented level of toxicity. The treated cells exhibited apoptotic features, including detachment from the culture site, condensation of the cytoplasm, shrinking of the cells, buildup of nuclear chromatin, and disruption of contact between adjacent cells. In addition, after treatment using Hydroalcoholic extract from *E. acoroides*, the number of viable cells tended to decrease in all dose groups [18].

Prior investigations have demonstrated a positive correlation between the findings of this study and the presence of anticancer activities in *E. acoroides* metabolites. This study utilizes both in silico and in vitro approaches to investigate the scavenging activity of different metabolite components originating from hexane or ethanol extraction procedures. Trolox (6-hydroxy-2,5,7,8-tetramethylchroman-2-carboxylic acid) in this study was used as a control component. Trolox is an analog of vitamin E which is hydrophilic and can act as an antioxidant because it can reduce the degree of damage and oxidative stress [19,20]. The experimental results indicate that there is no statistically significant distinction between EAE and Trolox when utilized as a control in the two-way ANOVA statistical analysis. This suggests that EAE possesses a scavenging capacity for oxidants, such as reactive oxygen species (ROS) (Figure 4), comparable to that of Trolox. On the other hand, the scavenging ability of EAH at various doses showed performance that was not significantly better when compared to controls except at a dose of 20.00 µg/dL. The results of this study show that the metabolite components obtained through extraction using hexane have lower ROS scavenging capabilities compared to EAE.

Oxidative stress is an important component that links toxicity in the surrounding environment to carcinogenic processes. Oxidative stress accumulation leads to damage in macromolecular components, including DNA, lipids, and proteins. Furthermore, oxidative lesions were implicated as one of the causes of cancer (Figure 4). ROS can facilitate an indirect assault on DNA by interacting with other biological components, particularly phospholipids [6,21,22]. This interaction leads to the production of different substances that can ultimately bind permanently to nitrogen bases in DNA. As a result, this causes a mismatch in the base pairs and disrupts the regular structure of DNA. Abnormalities in the formation and sequence of DNA are exacerbated by insufficiency in the DNA repair process so that errors in the DNA sequence accumulate every time the DNA is replicated during the mitosis process. When at one point this error changes the protooncogene into an oncogene, cancer cells begin to appear and proliferate uncontrollably.

Multiple studies indicate a correlation between cancer patients and the presence of low amounts of antioxidants and heightened oxidative stress. Recent research has indicated that individuals suffering from cancer exhibit diminished levels of vitamin C, vitamin E, glutathione, superoxide dismutase, and glutathione peroxidase in comparison to control groups or individuals in good health [6,22]. Conversely, cancer patients exhibited elevated MDA levels in comparison to healthy individuals. Antioxidant chemicals are alternatively referred to as radical-chain breakers (Figure 4). Antioxidant chemicals, like EAE and EAH, can undergo reactions with free radicals, such as peroxide radicals and singlet molecular oxygen. Antioxidants can interact with lipid peroxyl radicals, resulting in the stabilization of lipid hydroperoxide and the prevention of lipid peroxidation. The development of cancer is influenced by lipid peroxidation, as higher levels of lipid peroxidation lead to increased activity of the 15-lipooxygenases isoform 1 (15-LOX-1) [21]. Elevated 15-LOX-1 activity will trigger a cascade of chemical reactions that will indirectly impact the occurrence of errors in the pairing of DNA bases, hence initiating the development of oncogenes.

This study also conducted testing to assess the safety level of extract in *E. acoroides*. The LD_50_ value of the extracted sample, when tested on breast cancer cells and normal epithelial cells, was compared to that of doxorubicin as a control. The results indicate that the LD_50_ value of EAH and EAE in the breast cancer cell line is significantly greater than that of the control. This elucidates that a greater dosage of EAE and EAH is required in comparison to doxorubicin to achieve cytotoxic levels in cells. *E. acoroides*’ metabolite components have a high level of safety, rendering it a possible candidate for future chemotherapeutic alternatives. Moreover, this research presents a successful study that has thoroughly identified substances that suppress breast cancer from Seagrass *E. acoroides*. This was achieved by combining two methods: in silico molecular docking and in vitro validation. It is worth noting that both approaches have not been previously documented in any publication. The identification of chemicals in *E. acoroides* and their molecular actions will enhance our understanding of novel materials for combating cancer and serve as valuable references for further advanced research. Nevertheless, this research must be extended to further stages, including in vivo investigations on experimental animals and clinical trials on humans, to further evaluate its efficacy. Furthermore, it is imperative to separate each identified chemical that exhibits anti-cancer properties using computational analysis to advance its development as an individual product and evaluate its efficacy through in vivo experimentation.

## 4. Materials and Methods

### 4.1. Enhalus acoroides (EA) Extract Preparation and Metabolites Profiling

**Figure 5:**
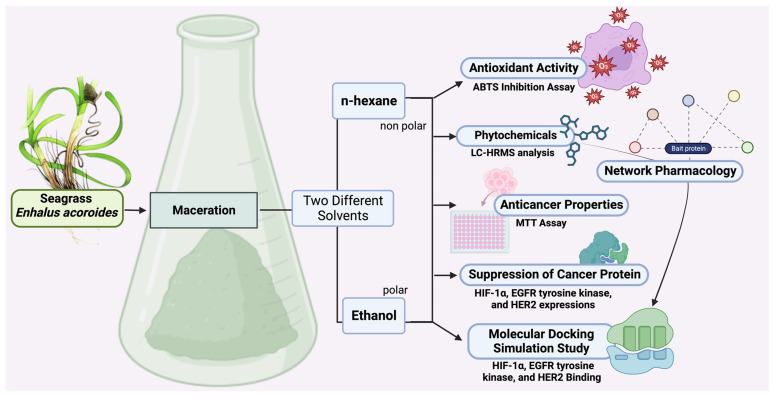
Methodical schematic of EA study flow. Created with BioRender.com Premium License by Fahrul Nurkolis (https://app.biorender.com, accessed on 5 January 2024).

*Enhalus acoroides* (EA) samples were collected from the waters of the North Sea of Central Java. Botanical identification and authentication of the samples were conducted by researchers at a laboratory in Indonesia and verified by the National Center for Biotechnology Information (NCBI) Taxonomy ID 55455 (NCBI: txid55455) database. The EA samples underwent a process of washing with distilled water and cleaning, followed by drying in a Memmert Incubator IN55 oven at a temperature of 50 °C for a duration of 72 h. Subsequently, the sample size was decreased by employing a Cosmos Blender 2 L ReBlend High-Speed Hand Blender, resulting in the production of coarse simplica powder. The simplicia powder was continued with extraction through maceration, a total of 400 g of EA simplicia powder was macerated using 4 L of 96% ethanol (C_2_H_5_OH) solvent for 72 h with occasional shaking. Subsequently, the filtrate undergoes another round of filtration, followed by a process of remaceration. Subsequently, the filtrate is consolidated and subjected to evaporation using a rotatory evaporator at a precisely controlled temperature of 50 °C. This is then followed by immersion in a water bath to generate a concentrated EA extract, which is further separated into fractions using the n-hexane solvent, Figure 5. Subsequently, both EAE and EAH extracts were preserved in aluminum foil for subsequent testing. This extraction approach pertains to analogous research that has been previously published [23].

Each sample was combined with 96% ethanol in a volume of 50 µL and then exposed to 30 vortex cycles. Subsequently, a centrifugation procedure was conducted, lasting 2 min at a velocity of 6000 rotations per minute (rpm). Prior to conducting the investigation, the supernatant underwent filtration using a 0.22 µm syringe filter. The investigation employed a Thermo Scientific Dionex Ultimate 3000 RSLC Nano HPLC system, which included a micro flow meter. Reconnaissance operations were conducted utilizing a Hypersil GOLD aQ 50 column with dimensions of 50 mm in length and 1 mm in diameter. The column had a particle size of 1.9 µm and was maintained at a temperature of 30 °C. This technique utilizes two solvents: Solvent A, which is composed of water with 0.1% formic acid, and Solvent B, which is composed of acetonitrile. The separation of compounds was performed by employing a linear gradient with a flow rate of 40 µL/min for a duration of 30 min. Precise instrumentation produced by Thermo Scientific was utilized to conduct high-resolution mass spectrometry (HRMS). The instrument demonstrates a scanning resolution of 70,000 for both positive and negative ionization modes, encompassing a wide range of measurements. In addition, it possesses a data-dependent MS2 resolution of 17,500.

### 4.2. In Silico Study Assessment

#### 4.2.1. Prediction of Bioactive Compound Activities, Toxicity Analysis, and Drug Likeness

Observed compounds from Seagrass *E. acoroides* were analyzed for potential bioactivity using the WAY2DRUG PASS prediction tool (http://www.pharmaexpert.ru/passonline/predict.php, accessed on 20 January 2024) for cancer treatment, which specifically targets breast cancer through SAR analysis to compare input compounds with known compounds that show specific potency [24]. The Pa value (probability of being active) represents the output prediction score obtained from the web, which shows the potency of the compound being tested and a Pa value > 0.7 indicates that the compound is predicted to have high potential, for example as an anticancer agent, because of its similarity to compounds in the database. Because the Pa value reflects the accuracy of the prediction function obtained, where a higher Pa value indicates greater accuracy, the Pa value used in the study is limited to >0.7. Furthermore, toxicity and drug likeness analysis represent a series of pharmacokinetic parameters that are important in drug development, assessing the potential toxicity effects of a drug. Drug similarity characteristics were determined for each ligand based on Lipinski’s Rule 5 (Ro5), which was analyzed using the Protox II database (https://tox-new.charite.de/protox_II/index.php?site=compound_input, accessed on 20 January 2024) and the ADMETLab 2.0 database (https://admetmesh.scbdd.com/service/evaluation/index, accessed on 20 January 2024) using the SMILES notation of each compound as input [25,26,27]. The SMILES notation for each compound was obtained from PubChem (https://pubchem.ncbi.nlm.nih.gov, accessed on 20 January 2024) and the data can be seen in Supplementary Table S2.

#### 4.2.2. Protein Target Identification and Analysis

Target analysis of Seagrass *E. acoroides* Extract was carried out using the SuperPred target analysis tool (https://prediction.charite.de/, accessed on 20 January 2024) by entering the SMILES notation for each compound (Table S1) and the cut-off score for SuperPred Target for the model’s probability and accuracy were set at 80% (range from 0 to 100%) [28,29]. Genes and proteins associated with breast cancer were taken from the Open Targets database (http://www.opentargets.org/, accessed on 20 January 2024). The disease-related targets and targets of Seagrass *E. acoroides* Extract were then mapped using a Venn diagram to determine the intersection of the corresponding targets. Target annotation of Seagrass *E. acoroides* Extract was carried out using the DAVID webserver (https://david.ncifcrf.gov/, accessed on 20 January 2024) with a focus on biological processes and Kyoto Encyclopedia of Genes and Genomes (KEGG) pathways [30].

#### 4.2.3. Network Pharmacology Analysis

Analysis of interactions between target proteins obtained from Seagrass *E. acoroides* Extract and their relationship to breast cancer was carried out using the STRING Database (Search Tool for Retrieval of Interacting Genes/Proteins) Version 12.0 [31]. The input consists of target proteins derived from Seagrass *E. acoroides* Extract along with intersections of proteins related to breast cancer carried out using the STRING Database (Search Tool for Retrieval of Genes/Proteins, including the HIF1A and Chlordecone reductase receptors which are known to be closely related to the incidence of breast cancer. In the analysis using the STRING Database, the organism was set as Homo sapiens (human), and to ensure strong interactions a high confidence score threshold of 0.9 was applied for this analysis. The resulting analysis data are presented in TSV format from the STRING database and downloaded to be processed for advanced analysis using CytoScape Version 3.10.1 for in-depth investigation of network analysis which also allows exploration of key network parameters such as degree, betweenness centrality, and closeness centrality between receptors [32].

#### 4.2.4. Molecular Docking Simulation

The docking simulation was conducted using cavity-detection-guided Blind Docking, specifically with CB-Dock2, an improved version of the CB-Dock server for protein–ligand blind docking. This method integrates cavity detection, docking, and homologous template fitting. The docking protocol followed the procedures outlined in previous publications [33,34]. CB-Dock2 is a protein–ligand docking method that automatically identifies binding sites, calculates their center and size, customizes the docking box size according to the query ligands, and performs molecular docking with AutoDock Vina. CB-Dock facilitates the docking procedure and improves accuracy by predicting the binding sites of target proteins using the curvature-based cavity detection approach (CurPocket) and the binding poses of query ligands using AutoDock Vina. For more detailed information and methodology, refer to the articles [33,34]. Furthermore, the observed receptors with the highest degree of centrality are used for further analysis in molecular docking, including receptors observed to be associated with their signaling pathways. 

The enzymes or proteins used were HIF-1α PDB ID: 3KCX; EGFR tyrosine kinase PDB ID: 1M17; HER2 PDB ID: 3PP0. Water molecules and other heteroatoms were deleted from the uploaded protein structures prior to docking by default by the CB2-Dock Sever. All receptor or target proteins.pdb format from RSCB Protein Data Bank (https://www.rcsb.org; accessed on 20 January 2024); Ligands were obtained from PubChem in .sdf form (https://pubchem.ncbi.nlm.nih.gov; accessed on 20 January 2024), and compounds not found in PubChem were visualized using 22.2.0 ChemDraw MacBook Version.

### 4.3. Antioxidant Capacity of EA against ABTS

Scavenging of 2,2′-Azino-bis (3-ethylbenzothiazoline-6-sulfonic acid) or diammonium salt radical cations ([ABTS+, C_18_H_24_N_6_O_6_S_4_] Sigma-Aldrich, Darmstadt, Germany) is determined by procedure by Hayes et al., (2023) and Sabrina et al., (2022) [35,36]. In total, 2.4 mM of Potassium persulfate (K_2_S_2_O_8_,) and 7 mM ABTS were mixed in a 1:1 ratio, protected from light with aluminum foil, and allowed to react at 22 °C for 14 h in dark conditions. The mixture is further diluted (e.g., 1 mL of stock solution plus 60 mL of EtOH (C_2_H_6_O) to obtain a working solution with an absorption of 0.706 at 734 nm. A new working solution is prepared for each test. The samples (EAE and EAH) were stored in gradients of 20, 40, 60, 80, and 100 μg/mL, respectively, to be diluted with ABTS working solution (1 mL), and absorbance was measured after 7 min at 734 nm. The inhibition of DPPH and ABTS is expressed as a percentage (%), and is determined according to the formula below:

(1) $$Inhibition\;Activity\;\left(\%\right)=\left[\frac{A0-A1}{A0}\right]\times 100\%\;$$

where A0 is the blank absorption, and A1 represents the standard or sample absorption.

### 4.4. In Vitro Study on Cancer Cell Lines

The American Type Culture Collection (ATCC; Manassas, VA, USA) supplied cell lines for Human breast cancer cells (MCF-7 cell lines ATCC^®^ no. HTB-22™, MDA-MB-231 ATCC^®^ no. HTB-26™) and normal breast epithelial (MCF-10A cell lines ATCC^®^ no. CRL-10317) in the Biochemistry and Biomolecular Laboratory of the Faculty of Medicine Universitas Brawijaya (Malang, Indonesia). MCF-7, MDA-MB-231, and MCF-10A cells (1 × 10^5^) were cultured in 96 well plates containing DMEM (Dulbecco’s Modified Eagle Medium; Thermo Fisher Scientific, Waltham, MA, USA) with 10% Fetal Bovine Serum (FBS; Thermo Fisher Scientific, USA) and 1% antibiotics (100 UI/mL-Penicillin and 100 μL/mL-Streptomycin), refer to the manufacturer’s protocol. Once the cultured cells reach 80% density, the cells are incubated in an incubator with 5% CO_2_ at 37 °C. Cells are harvested periodically using a solution of trypsin-ethylenediaminetetraacetic acid (trypsin-EDTA; Thermo Fisher Scientific, USA).

#### 4.4.1. Antiproliferative Activity of EA via MTT Assay

The cytotoxicity test of human breast cancer cells and also normal breast cell lines MCF-10A was carried out by the MTT method according to the method Nurkolis et al., (2023) [23]. Prepare MCF-7, MDA-MB-231, and MCF-10A breast cancer cells incubated on 96 well plates for 24 h. MCF-7, MDA-MB-231, and MCF-10A cells were administered with EAE and EAH at concentrations of 0, 50, 100, 150, 200, and 250 μg/mL, and doxorubicin (positive controls; Sigma-Aldrich, Darmstadt, Germany) were also treated similarly with reference to similar studies. EAE, EAH, Luteolin (L9283-10MG, Sigma-Aldrich, Darmstadt, Germany) and Thalassiolin C (518057-56-2), and doxorubicin were added and incubated for 24 h. After that, the cells were isolated with 1X PBS liquid and incubated with 100 μL MTT 0.5 mg/mL at 37 °C. After 30 min, 100 μL of DMEM stopper reagent was added to each well plate. Absorbance was measured at a wavelength of 550 nm using a microplate reader. To minimize the risk of bias, three triple trials were performed for each treatment group. The eligible cells are presented as percentages with the formula mentioned below:

(2) $$Percentage\;of\;Living\;Cells\;or\;Viability\;\left(\%\right)=\frac{A-B}{C-B}\;$$

Description = A: Cell absorbance with treatment; B: Absorbance of blank samples; C: Control cell absorbance.

#### 4.4.2. HIF-1α, EGFR Tyrosine Kinase, and HER2 Expressions

In vitro analysis of HIF-1α, EGFR tyrosine kinase, and HER2 Expressions was carried out in accordance with the manufacturer’s protocol (HIF-1 alpha Monoclonal Antibody (ESEE122), eBioscience™; Human EGFR (Epidermal Growth Factor Receptor) ELISA Kit; Elabscience^®^ for HER2) and established research experimental guidelines [37]. To detect HIF-1α, EGFR tyrosine kinase, and HER2, the polyvinylidene difluoride membrane was treated with a blocking solution consisting of 5% skimmed dry milk in a buffer consisting of Tris with Tween (T-TBS) saline buffer. This is performed to prevent the membrane from absorbing any detection reagents. This buffer has a concentration of 0.1% Tween 20 and contains 20 mmol/L Tris-HCl, 0.138 mol/L Sodium chloride (NaCl; Sigma-Aldrich, Darmstadt, Germany), and has a pH of 7.4. On the other hand, to identify phosphorylated HIF-1α, EGFR tyrosine kinase, and HER2, a blocking solution consisting of 5% albumin (specifically bovine serum albumin or BSA) in T-TBS is used to treat the membrane. This is performed so that phosphorylated HIF-1α, EGFR tyrosine kinase, and HER2 can be detected. To assess the expression of HIF-1α, EGFR tyrosine kinase, and HER2, a special methodology was followed. The process includes exposing the cell membrane to primary antibodies, followed by secondary antibodies associated with peroxidase. Primary and secondary antibodies were diluted in a solution containing 5% Bovine Serum Albumin (BSA) in a T-TBS solution. By adopting this comprehensive antibody-based technique, the study aims to gain insight into HIF-1α, EGFR tyrosine kinase, and HER2 expressions, while ensuring precision through antibody dilution and appropriate incubation conditions. To complete the information, the experimental process involved seeding 5000 MCF-7 cells into each well using 100 μL/well. These cells were treated with Seagrass *E. acoroides* Extract with different concentration gradients of 0, 50, 100, 150, 200, and 250 μg/mL within a 24-h incubation time. Next, the data obtained were analyzed to ascertain the percentage value relative to the control group (a group consisting of cells that were not given any treatment or 0 μg/mL of Seagrass *E. acoroides* Extract). This percentage (%) value assessment is facilitated through optical density (OD) measurements performed using spectrophotometers (SmartSpec Plus from Bio-Rad Laboratories. Inc., Hercules, CA, USA) at wavelengths of 665 and 620 nm.

### 4.5. Data Management and Analysis

Statistical analysis of data was carried out using the MacBook version of GraphPad Prism Premium 10 software (GraphPad Software, Inc.; San Diego, CA, USA). The Shapiro–Wilk test is performed to evaluate the distribution of data. If the data were normally distributed (significance < 0.05), a One-Way ANOVA test was performed to test the average difference between treatment groups. Otherwise, the Kruskal–Wallis test will be performed. The lethal value of 50% (Lethal Concentration 50 or LC_50_) of breast cancer cells and antioxidant activity (ABTS) were analyzed using the statistical analysis package GraphPad Premium ‘non-linear regression (log(inhibitor) vs. normalized response-variable slope’ while seeing the significance value (95%CI) of antioxidant activity via Two-Way ANOVA test.

## 5. Conclusions

This research successfully profiled the metabolites of Seagrass *Enhalus acoroides* through both in silico and in vitro studies, revealing its ability to combat breast cancer by inhibiting HIF-1α, EGFR tyrosine kinase, and HER2 through molecular docking. Furthermore, the subsequent in vitro investigation revealed new evidence that Seagrass *E. acoroides*-ethanol (EAE) is highly potent in fighting breast cancer. The comprehensive combination of these two approaches identified a promising new source of natural materials that can be studied and developed at a further stage. Notably, EAE demonstrated more potent free radical-fighting activity than Trolox as a control antioxidant. Therefore, future research should involve in vivo and human clinical trials to further evaluate the efficacy of EAE, concerning the doses reported in this study.

## Figures and Tables

**Table 1:** Metabolites observed in Seagrass *E. acoroides* via HPLC-ESI-HRMS/MS analysis.

Samples	No	Observed Compounds	Molecular Formula	RT (min)	Observed MW (*m*/*z*)	PubChem ID or Substance ID
EAE	C1	Thalassiolin A	C_21_H_20_O_14_S	6.50	528.4200	5493604
	C2	Luteolin	C_15_H_10_O_6_	12.91	286.1500	5280445
	C3	luteolin-O-sulphate	C_15_H_10_O_9_S	10.25	366.2500	NA
	C4	Myricetin	C_15_H_10_O_8_	20.33	317.9189	5281672
	C5	di-O-caffeoyl tartaric acid	C_22_H_18_O_12_	7.15	473.0370	NA
EAH	C6	6-hydroxy luteolin *O*-glucoside	C_21_H_20_O_12_	10.91	464.1050	185766
	C7	Oleamide	C_18_H_35_NO	3.33	281.3700	5283387
	C8	Thalassiolin C	C_21_H_20_O_13_S	7.21	512.5400	5493606
	C9	*O*-caffeoyl-*O*-coumaroyl tartaric acid	C_22_H_18_O_11_	9.10	458.0908	NA
	C10	Betaine	C_5_H_11_NO_2_	11.73	117.0135	247

EAE: *E. acoroides*—ethanol (polar); EAH: *E. acoroides*—hexane (non-polar). RT: Retention Time (Minutes); MW: Molecular Weight; NA: Not Applicable.

**Table 2:** The evaluation of *E. acoroides* potential for anticancer based on structure–activity relationship (SAR) predictions, Pa Score, Toxicity Prediction, Drug Likeness and Network Pharmacology Analysis.

Compounds	Pa Score *		Toxicity Model Computation Analysis **		Drug Likeness ***		
	HIF1A Expression Inhibitor	Chlordecone Reductase Inhibitor	Predicted LD_50_ (mg/kg)	Toxicity Class	Lipinski Rule	Pfizer Rule	GSK
C1	0.80	0.46	5000	5	Rejected	Accepted	Rejected
C2	0.96	0.98	3919	5	Accepted	Accepted	Accepted
C3	0.90	0.915	4000	5	Accepted	Accepted	Accepted
C4	0.97	0.99	159	3	Accepted	Accepted	Accepted
C5	0.76	0.87	2980	5	Rejected	Accepted	Rejected
C6	0.84	0.71	5000	5	Rejected	Accepted	Rejected
C7	0.14	0.55	750	4	Accepted	Rejected	Rejected
C8	0.79	0.41	5000	5	Rejected	Accepted	Rejected
C9	0.58	0.60	650	4	Accepted	Accepted	Rejected
C10	0.12	0.72	650	4	Accepted	Accepted	Accepted

* Way2Drug; ** Protox; *** ADMET.

**Table 3:** Results of the top one protein–protein interaction (PPI) network analyses.

Name	Degree	Betweenness Centrality	Closeness Centrality	Overall Score	Pathway
EGFR	17	0.2315	0.4655	17.6970	Breast cancer, HIF-1 signaling pathway, EGFR tyrosine kinase inhibitor resistance (Cancer), ERK and HER signaling (cancer), and PI3K signaling (cancer)

**Table 4:** ΔG of Molecular docking parameter of identified compounds of Seagrass *E. acoroides*.

Compounds and Control as Ligands	HIF-1α	EGFR Tyrosine Kinase	HER2
Control Doxorubicin	−8.6	−7.2	−8.7
Control Talazoparib	−7.7	−7.9	−8.4
C2	−8.7	−8.1	−9.8
C3	−9.5	−8.4	−10.0
C8	−8.9	−8.3	−8.9
C9	−8.9	−8.3	−9.9

**Table 5:** Amino acid interaction visualization of identified compounds from Seagrass *E. acoroides* against Selected Receptors.

Ligands	HIF-1α 3KCX	EGFR Tyrosine Kinase 1M17	HER2 3PP0
Control Doxorubicin	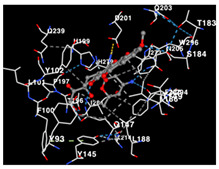	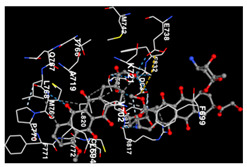	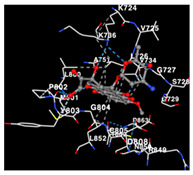
Control Talazoparib	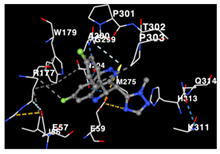	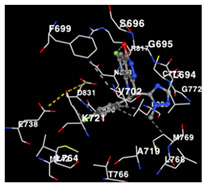	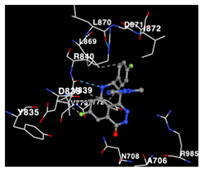
C2	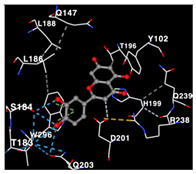	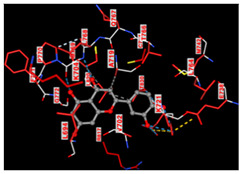	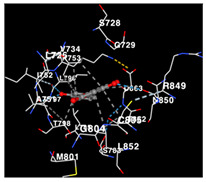
C3	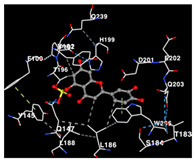	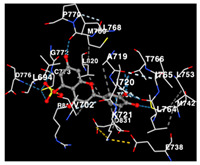	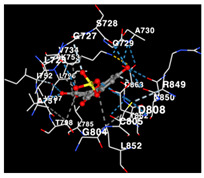
C8	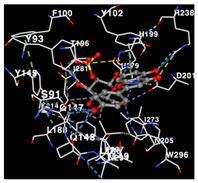	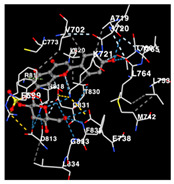	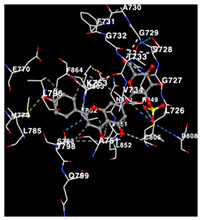
C9	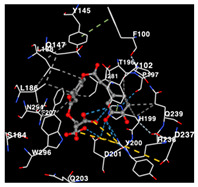	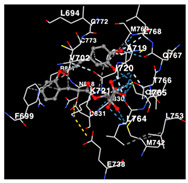	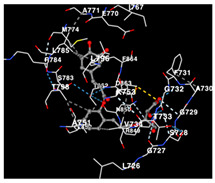

**Table 6:** LD_50_ Values (μg/mL) Exhibited by Seagrass *E. acoroides* on Breast Cancer Cell Lines and Normal Epithelial Cell Lines.

No	Samples	MCF-7	MDA-MB-231	Normal Cell (MCF-10A)
1	EAE	220.5650	550.8885	1780.2050
2	EAH	345.9544	1500.6800	1950.1045
3	Luteolin	101.0012	1201.5516	1500.2159
4	Thalassiolin C	100.1150	985.7500	1312.3460
3	Control Doxorubicin	3.1955	0.4455	54.0025

## Data Availability

The data presented in this study are available on request from the corresponding author.

## Supplementary Materials

The following supporting information can be downloaded at: https://www.mdpi.com/article/10.3390/molecules29051082/s1, Table S1: The values of degree, betweenness centrality, and closeness centrality results of the protein–protein interaction (PPI) network pharmacology analyses; Table S2: SMILES Canonical for from seagrass *Enhalus acoroides*.

## References

[1] Nur R.M., Nurafni, Koroy K., Alwi D., Wahab I., Sulistiawati S., Dewi R., Rorano M. (2021). The antibacterial activity of seagrass *Enhalus acoroides* against *Staphylococcus aureus*. Proceedings of the IOP Conference Series: Earth and Environmental Science.

[2] Gono C.M.P., Ahmadi P., Hertiani T., Septiana E., Putra M.Y., Chianese G. (2022). A Comprehensive Update on the Bioactive Compounds from Seagrasses. Mar. Drugs.

[3] Duffy J.E., Benedetti-Cecchi L., Trinanes J., Muller-Karger F.E., Ambo-Rappe R., Boström C., Buschmann A.H., Byrnes J., Coles R.G., Creed J. (2019). Toward a Coordinated Global Observing System for Seagrasses and Marine Macroalgae. Front. Mar. Sci..

[4] Hemminga M.A., Duarte C.M. (2000). Seagrass Ecology.

[5] Wahab I., Madduppa H., Kawaroe M. (2017). Seagrass species distribution, density and coverage at Panggang Island, Jakarta. Proceedings of the IOP Conference Series: Earth and Environmental Science.

[6] Kim D.H., Mahomoodally M.F., Sadeer N.B., Seok P.G., Zengin G., Palaniveloo K., Khalil A.A., Rauf A., Rengasamy K.R. (2021). Nutritional and bioactive potential of seagrasses: A review. S. Afr. J. Bot..

[7] Fatmawati Y., Sandrina S., Aina R.N., Narulita E. (2022). Molecular docking analysis of seagrass (*Enhalus acoroides*) phytochemical compounds as an antidiabetic. J. Biol. Res..

[8] Wang X.-B., Sun Z.-H., Fan L.-X., Liu Y.-Y., Feng J., Ma G.-X., Chen D.-L. (2021). Two novel diterpenes from the stems and leaves of tropical seagrass *Enhalus acoroides* in the South China sea. Nat. Prod. Res..

[9] Selim M.S.M., Abdelhamid S.A., Mohamed S.S. (2021). Secondary metabolites and biodiversity of actinomycetes. J. Genet. Eng. Biotechnol..

[10] El Shaffai A., Mettwally W.S.A., Mohamed S.I.A. (2023). A comparative study of the bioavailability of Red Sea seagrass, *Enhalus acoroides* (L.f.) Royle (leaves, roots, and rhizomes) as anticancer and antioxidant with preliminary phytochemical characterization using HPLC, FT-IR, and UPLC-ESI-TOF-MS spectroscopic. Beni-Suef Univ. J. Basic Appl. Sci..

[11] Sudo K., Nakaoka M. (2020). Fine-scale distribution of tropical seagrass beds in Southeast Asia. Ecol. Res..

[12] Fredotović Ž., Puizina J., Nazlić M., Maravić A., Ljubenkov I., Soldo B., Vuko E., Bajić D. (2021). Phytochemical Characterization and Screening of Antioxidant, Antimicrobial and Antiproliferative Properties of *Allium* × *cornutum* Clementi and Two Varieties of *Allium cepa* L. Peel Extracts. Plants.

[13] De Vincenti L., Glasenapp Y., Cattò C., Villa F., Cappitelli F., Papenbrock J. (2018). Hindering the formation and promoting the dispersion of medical biofilms: Non-lethal effects of seagrass extracts. BMC Complement. Altern. Med. Ther..

[14] Sritharan S., Sivalingam N. (2021). A comprehensive review on time-tested anticancer drug doxorubicin. Life Sci..

[15] Renu K., Abilash V.G., Tirupathi T.P., Arunachalam S. (2018). Molecular mechanism of doxorubicin-induced cardiomyopathy—An update. Eur. J. Pharmacol..

[16] Chandel N.S. (2021). Basics of metabolic reactions. Cold Spring Harb. Perspect. Biol..

[17] Mabrouk S.B., Reis M., Sousa M.L., Ribeiro T., Almeida J.R., Pereira S., Antunes J., Rosa F., Vasconcelos V., Achour L. (2020). The Marine Seagrass *Halophila stipulacea* as a Source of Bioactive Metabolites against Obesity and Biofouling. Mar. Drugs.

[18] Ahmed N., Vasantha K.S., John A.K., Shobana C., Usharani B. (2022). Anticancer activity of hydroalcoholic extract of *Enhalus acoroides*. Int. J. Health Sci..

[19] Çelik E.E., Rubio J.M.A., Gökmen V. (2018). Behaviour of Trolox with macromolecule-bound antioxidants in aqueous medium: Inhibition of auto-regeneration mechanism. Food Chem..

[20] Harmankaya A., Özcan A., Dalginli K., Erdag D., Aydın Dursun Y., Gungor B. (2021). The Effect of Trolox on Oxidative Stress Index and Nitric Oxide Levels. J. Inst. Sci. Technol..

[21] Clemente S.M., Martínez-Costa O.H., Monsalve M., Samhan-Arias A.K. (2020). Targeting Lipid Peroxidation for Cancer Treatment. Molecules.

[22] Fuchs-Tarlovsky V. (2013). Role of antioxidants in cancer therapy. Nutrition.

[23] Nurkolis F., Taslim N.A., Qhabibi F.R., Kang S., Moon M., Choi J., Choi M., Park M.N., Mayulu N., Kim B. (2023). Ulvophyte Green Algae *Caulerpa lentillifera*: Metabolites Profile and Antioxidant, Anticancer, Anti-Obesity, and In Vitro Cytotoxicity Properties. Molecules.

[24] Druzhilovskiy D.S., Rudik A.V., Filimonov D.A., Gloriozova T.A., Lagunin A.A., Dmitriev A.V., Pogodin P.V., Dubovskaya V.I., Ivanov S.M., Tarasova O.A. (2017). Computational platform Way2Drug: From the prediction of biological activity to drug repurposing. Russ. Chem. Bull..

[25] Banerjee P., Eckert A.O., Schrey A.K., Preissner R. (2018). ProTox-II: A webserver for the prediction of toxicity of chemicals. Nucleic Acids Res..

[26] Norinder U., Bergström C.A.S. (2006). Prediction of ADMET properties. ChemMedChem.

[27] Dong J., Wang N.N., Yao Z.J., Zhang L., Cheng Y., Ouyang D., Lu A.P., Cao D.S. (2018). Admetlab: A platform for systematic ADMET evaluation based on a comprehensively collected ADMET database. J. Cheminform..

[28] Gallo K., Goede A., Preissner R., Gohlke B.-O. (2022). SuperPred 3.0: Drug classification and target prediction—A machine learning approach. Nucleic Acids Res..

[29] Dunkel M., Günther S., Ahmed J., Wittig B., Preissner R. (2008). SuperPred: Drug classification and target prediction. Nucleic Acids Res..

[30] Gfeller D., Grosdidier A., Wirth M., Daina A., Michielin O., Zoete V. (2014). SwissTargetPrediction: A web server for target prediction of bioactive small molecules. Nucleic Acids Res..

[31] Asadzadeh A., Ghorbani N., Dastan K. (2023). Identification of druggable hub genes and key pathways associated with cervical cancer by protein-protein interaction analysis: An in silico study. Int. J. Reprod. Biomed..

[32] Sun P., Yang Y., Cheng H., Fu S., Liu Y., Qiu Y., Chen H., Zhang J., Zhou H., Shi L. (2023). Integrated Analysis of Long Non-Coding RNA Expression Profiles in *Glaesserella parasuis*-Induced Meningitis: New Insight into Pathogenesis. Microbiol. Res..

[33] Liu Y., Yang X., Gan J., Chen S., Xiao Z.X., Cao Y. (2022). CB-Dock2: Improved protein-ligand blind docking by integrating cavity detection, docking and homologous template fitting. Nucleic Acids Res..

[34] Yang X., Liu Y., Gan J., Xiao Z.X., Cao Y. (2022). FitDock: Protein–ligand docking by template fitting. Brief. Bioinform..

[35] Hayes C., Nurkolis F., Laksemi D.A., Chung S., Park M.N., Choi M., Choi J., Darmaputra I.G., Gunawan W.B., Lele J.A. (2023). Coffee Silverskin Phytocompounds as a Novel Anti-Aging Functional Food: A Pharmacoinformatic Approach Combined with In Vitro Study. Molecules.

[36] Sabrina N., Rizal M., Nurkolis F., Hardinsyah H., Tanner M.J., Gunawan W.B., Handoko M.N., Mayulu N., Taslim N.A., Puspaningtyas D.S. (2022). Bioactive peptides identification and nutritional status ameliorating properties on malnourished rats of combined eel and soy-based tempe flour. Front. Nutr..

[37] Nurkolis F., Purnomo A.F., Alisaputra D., Gunawan W.B., Qhabibi F.R., Park W., Moon M., Taslim N.A., Park M.N., Kim B. (2023). In silico and in vitro studies reveal a synergistic potential source of novel anti-ageing from two Indonesian green algae. J. Funct. Foods.

