# Supplementary material for: Revealing Novel Source of Breast Cancer Inhibitors from Seagrass Enhalus acoroides: In Silico and In Vitro Studies
Source: Molecules. 2024 Feb 29;29(5):1082. doi: 10.3390/molecules29051082 (PMC10933956; doi:10.3390/molecules29051082)
Supplement: Supplementary file 1 [file molecules-29-01082-s001.zip › molecules-2891177-supplementary.pdf]

Supplementary Table S1. The values of degree, betweenness centrality, and closeness centrality results of the protein–protein interaction (PPI) network pharmacology analyses.

AverageShortestPathLength	BetweennessCentrality	ClosenessCentrality	ClusteringCoefficient	Degree	Eccentricity	IsSingleNode	name	NeighborhoodConnectivity	NumberOfDirectedEdges	NumberOfUndirectedEdges	PartnerOfMultiEdgedNodePairs	Radiality	selected	SelfLoops	shared name	Stress	TopologicalCoefficient	Name
4.03030303	0.27272727	0.2481203	0	3	7	FALSE	MAPT	2.33333333	0	3	0	0.76689977	FALSE	0	MAPT	368	0.33333333	ESR2
5.78787879	0	0.17277487	0	1	9	FALSE	BACE1	4	0	1	0	0.63170163	FALSE	0	BACE1	0	0	FLT3
1.66666667	0	0.6	1	3	3	FALSE	AURKB	4.33333333	0	3	0	0.86666667	FALSE	0	AURKB	0	0.86666667	GSK3B
1.5	0.02222222	0.66666667	0.83333333	4	3	FALSE	PLK1	3.75	0	4	0	0.9	FALSE	0	PLK1	2	0.75	HSD17B1
1.5	0.02222222	0.66666667	0.83333333	4	3	FALSE	TOP2A	3.75	0	4	0	0.9	FALSE	0	TOP2A	2	0.75	HSD17B2
1.16666667	0.55555556	0.85714286	0.5	5	2	FALSE	CDK1	3.2	0	5	0	0.96666667	FALSE	0	CDK1	18	0.6	IGF1R
3.33333333	0	0.3	0	1	6	FALSE	AXL	13	0	1	0	0.82051282	FALSE	0	AXL	0	0	INSR
5	0	0.2	0	1	8	FALSE	CAMK2B	3	0	1	0	0.69230769	FALSE	0	CAMK2B	0	0	KDR
1.66666667	0.33333333	0.6	0	2	2	FALSE	CDK2	3	0	2	0	0.86666667	FALSE	0	CDK2	10	0.5	MAPT
1.66666667	0	0.6	1	3	3	FALSE	NEK2	4.33333333	0	3	0	0.86666667	FALSE	0	NEK2	0	0.86666667	MET
2.5	0	0.4	0	1	3	FALSE	CDK6	2	0	1	0	0.7	FALSE	0	CDK6	0	0	MMP2
2.84848485	0.37878788	0.35106383	0	2	6	FALSE	ESR2	8.5	0	2	0	0.85780886	FALSE	0	ESR2	520	0.5	MMP3
3.12121212	0.00315657	0.32038835	0.8	5	6	FALSE	EGFR	7.8	0	5	0	0.83682984	FALSE	0	EGFR	12	0.4875	MMP9
3.12121212	0.01704545	0.32038835	0.7	5	6	FALSE	IGF1R	7.6	0	5	0	0.83682984	FALSE	0	IGF1R	44	0.475	MPO
3.27272727	0	0.30555556	1	3	6	FALSE	MET	8.33333333	0	3	0	0.82517483	FALSE	0	MET	0	0.64102564	NEK2
3.06060606	0.00820707	0.32673267	0.57142857	7	6	FALSE	PTK2	6.28571429	0	7	0	0.84149184	FALSE	0	PTK2	26	0.39285714	PARP1
3.6969697	0	0.2704918	0	1	6	FALSE	FLT3	11	0	1	0	0.79254079	FALSE	0	FLT3	0	0	PIK3CG
3.66666667	0	0.27272727	1	2	6	FALSE	INSR	8	0	2	0	0.79487179	FALSE	0	INSR	0	0.72727273	PIK3R1
3.18181818	0	0.31428571	1	3	6	FALSE	KDR	10.3333333	0	3	0	0.83216783	FALSE	0	KDR	0	0.64583333	PLK1
3.24242424	0	0.30841121	1	2	6	FALSE	MMP2	8.5	0	2	0	0.82750583	FALSE	0	MMP2	0	0.56666667	PTK2
3.18181818	0.11931818	0.31428571	0.16666667	4	6	FALSE	MMP9	4.25	0	4	0	0.83216783	FALSE	0	MMP9	138	0.28846154	SRC
4.15151515	0	0.24087591	0	1	7	FALSE	MMP3	4	0	1	0	0.75757576	FALSE	0	MMP3	0	0	SYK
4.15151515	0	0.24087591	0	1	7	FALSE	MPO	4	0	1	0	0.75757576	FALSE	0	MPO	0	0	TOP2A
3.21212121	0	0.31132075	1	2	6	FALSE	SYK	12	0	2	0	0.82983683	FALSE	0	SYK	0	0.75	TTR

Supplementary Table S2. SMILES Canonical for from seagrass *Enhalus acoroides*

Samples	NO	Compounds	SMILES Canonical
EAE	C1	Thalassiolin A	C1=CC(=C(C=C1C2=CC(=O)C3=C(C=C(C=C3O2)OC4C(C(C(C(O4)CO)O)O)OS(=O)(=O)O)O)O)O
	C2	Luteolin	C1AR=CC(=C(C=C1C2=CC(=O)C3=C(C=C(C=C3O2)O)O)O)O
	C3	luteolin-O-sulphate	O=C1C=C(C2=CC=C(O)C(O)=C2)OC3=C1C(OS(=O)(O)=O)=CC(O)=C3
	C4	Myricetin	C1=C(C=C(C(=C1O)O)O)C2=C(C(=O)C3=C(C=C(C=C3O2)O)O)O
	C5	di-O-caffeoyl tartaric acid	O=C(O)C(C(C(O)=O)OOC/C=C/C1=CC=C(O)C(O)=C1)OOC/C=C/C2=CC=C(O)C(O)=C2
EAH	C6	6-hydroxy luteolin *O*-glucoside	C1=CC(=C(C=C1C2=CC(=O)C3=C(C(=C(C=C3O2)OC4C(C(C(C(O4)CO)O)O)O)O)O)O)O
	C7	Oleamide	CCCCCCCCC=CCCCCCCCC(=O)N
	C8	Thalassiolin C	C1=CC(=CC=C1C2=CC(=O)C3=C(C=C(C=C3O2)OC4C(C(C(C(O4)CO)O)O)OS(=O)(=O)O)O)O
	C9	*O*-caffeoyl-*O*-coumaroyl tartaric acid	OC(C(O)=O)C(OC(/C=C/C1=CC=CC=C1OC(/C=C/C2=CC=C(O)C(O)=C2)=O)=O)C(O)=O
	C10	Betaine	C[N+](C)(C)CC(=O)[O-]
